# Median Arcuate Ligament Syndrome: Diagnostic Challenges and Outcomes Following Laparoscopic Decompression

**DOI:** 10.7759/cureus.112894

**Published:** 2026-07-18

**Authors:** Rajesh K Nanjundaiah, Pallavi H Raghavendra, Naveen Narayan, Kruthi Nataraj, Sindhu D Nagaraj

**Affiliations:** 1 General Surgery, Adichunchanagiri Institute of Medical Sciences, Balagangadharanatha Nagara, IND; 2 Plastic Reconstructive and Aesthetic Surgery, Adichunchanagiri Institute of Medical Sciences, Balagangadharanatha Nagara, IND; 3 General Surgery, Adichunchanagiri Hospital and Research Centre, Balagangadharanatha Nagara, IND

**Keywords:** celiac artery compression, chronic abdominal pain, laparoscopic decompression, median arcuate ligament syndrome, retrospective study, visceral ischemia

## Abstract

Background and aim: Median arcuate ligament syndrome (MALS) is an uncommon vascular compression disorder caused by extrinsic compression of the celiac artery and surrounding neural plexus by the median arcuate ligament. Diagnosis is frequently delayed because of nonspecific symptoms and the presence of incidental radiological celiac artery compression in asymptomatic individuals. By providing detailed clinicoradiological correlation and operative outcome data, this study aimed to contribute to the growing body of evidence regarding the diagnosis and management of this uncommon but clinically significant disorder.

Methods: This retrospective cohort study was conducted at a tertiary care teaching hospital to evaluate the clinical presentation, radiological findings, operative characteristics, and postoperative outcomes of patients undergoing laparoscopic median arcuate ligament release between January 2024 and December 2025. Demographic, clinical, radiological, operative, and follow-up data were analyzed. Computed tomography angiography (CTA) was used to assess celiac artery compression and associated vascular changes. Clinical success was defined as complete resolution or substantial improvement of symptoms without requirement for reintervention.

Results: A total of 10 patients underwent laparoscopic median arcuate ligament release and met all inclusion criteria. The mean age was 36.5±16.8 years, and six patients (60%) were male. The median duration of symptoms prior to diagnosis was 18 months (range: 4-72 months). Postprandial abdominal pain was the predominant presenting symptom, occurring in 90% of patients. CTA demonstrated characteristic celiac artery compression in all patients, with severe stenosis (>70%) identified in seven patients (70%). All procedures were completed laparoscopically without conversion to open surgery. Mean operative time was 118±24 min, and mean hospital stay was 5.2±2.1 days. One patient presented with severe gastric ischemia requiring concomitant gastric fundal resection. At a median follow-up of 12 months, complete symptom resolution was achieved in six patients (60%), while three patients (30%) experienced significant symptomatic improvement, resulting in an overall favorable outcome rate of 90% (95% CI: 55.5-99.7%). Mean visual analog scale pain scores improved from 8.1±0.7 preoperatively to 2.3±1.3 at follow-up.

Conclusions: MALS remains an underrecognized cause of chronic postprandial abdominal pain associated with substantial diagnostic delay. Careful clinicoradiological assessment and multidisciplinary evaluation facilitate accurate diagnosis and appropriate patient selection. Laparoscopic median arcuate ligament release is a safe and effective treatment strategy, providing durable symptom relief in the majority of patients. The occurrence of severe gastric ischemia in one patient underscores the potential consequences of delayed diagnosis. Larger prospective multicenter studies are required to validate radiological severity classifications and identify predictors of surgical success.

## Introduction

Median arcuate ligament syndrome (MALS), also referred to as celiac artery compression syndrome or Dunbar syndrome, is a rare and frequently underrecognized vascular compression disorder caused by extrinsic compression of the proximal celiac artery and adjacent celiac plexus by the median arcuate ligament, a fibrous arch connecting the diaphragmatic crura across the aortic hiatus [1,2]. Although anatomical compression of the celiac artery may be identified in up to 10-24% of asymptomatic individuals on imaging studies, only a small proportion develop clinically significant symptoms, making the diagnosis particularly challenging and often controversial [2-4].

The syndrome most commonly affects young and middle-aged individuals and has historically been reported more frequently in females; however, symptomatic disease can occur across all age groups and both sexes [2,5]. Patients typically present with chronic postprandial epigastric pain, nausea, vomiting, early satiety, abdominal bloating, gastroesophageal reflux symptoms, food avoidance, and progressive weight loss [3,5]. These manifestations overlap substantially with numerous gastrointestinal, hepatobiliary, pancreatic, and functional disorders, often resulting in extensive investigations and prolonged diagnostic delays before MALS is considered [6]. Consequently, many patients undergo multiple consultations and diagnostic procedures before a definitive diagnosis is established.

Despite decades of investigation, the exact pathophysiological basis of MALS remains incompletely understood. Traditionally, symptoms were attributed to foregut ischemia resulting from compromised blood flow through the celiac artery during expiration, when diaphragmatic elevation accentuates vascular compression [3,7]. However, the relatively rich collateral circulation between the celiac artery and superior mesenteric artery challenges the notion that ischemia alone is responsible for symptom development [8]. Increasing evidence suggests that irritation and chronic stimulation of the celiac ganglion and perivascular sympathetic nerve plexus may play a significant role in generating neuropathic abdominal pain [6,9]. Contemporary understanding therefore supports a multifactorial mechanism involving both vascular insufficiency and neurogenic dysfunction, which may explain the poor correlation frequently observed between radiological severity and clinical symptom burden [6,9].

The diagnosis of MALS requires careful integration of clinical presentation and radiological findings. Computed tomography angiography (CTA) has emerged as the imaging modality of choice owing to its ability to demonstrate characteristic focal narrowing of the proximal celiac artery, hooked or J-shaped configuration of the vessel, poststenotic dilatation, and associated collateral circulation [3,10]. Duplex Doppler ultrasonography may demonstrate elevated expiratory peak systolic velocities and dynamic respiratory variation, while magnetic resonance angiography offers additional anatomical assessment in selected patients [11]. Nevertheless, imaging findings alone are insufficient for diagnosis because similar anatomical compression can be observed in asymptomatic individuals. Consequently, MALS remains a diagnosis of exclusion that requires thorough evaluation to eliminate alternative gastrointestinal, vascular, and functional causes of chronic abdominal pain [2,6].

Management strategies for MALS have evolved considerably over recent decades. While endovascular interventions may address residual vascular stenosis, they fail to relieve the underlying extrinsic compression and associated neural irritation, limiting their role as primary treatment modalities [12]. Surgical decompression through division of the median arcuate ligament remains the cornerstone of management in carefully selected symptomatic patients [5,12]. Historically performed through an open approach, treatment has increasingly shifted toward minimally invasive laparoscopic and robotic techniques, which provide excellent visualization of the celiac axis while reducing perioperative morbidity, postoperative pain, and hospital stay [5,13]. Several contemporary studies have reported symptom improvement in 60-90% of patients following surgical release, although outcome variability remains substantial and predictors of long-term success are not fully understood [5,13,14].

A further challenge in understanding MALS lies in its rarity and the predominance of published evidence from small retrospective series, primarily originating in North America and Europe. Consequently, significant gaps remain regarding the clinical presentation, radiological characteristics, operative findings, and surgical outcomes of patients treated in other geographical regions, particularly within South Asia. Furthermore, uncommon manifestations of severe visceral ischemia remain sparsely documented in the literature despite their potentially life-threatening consequences [8,14].

Given these considerations, the present study was undertaken to evaluate the demographic characteristics, clinical spectrum, diagnostic challenges, radiological features, operative findings, and postoperative outcomes of patients undergoing laparoscopic median arcuate ligament release at a tertiary care teaching hospital. In addition to assessing overall surgical outcomes, this series highlights a rare presentation complicated by severe gastric ischemia requiring gastric resection.

## Materials and methods

Study design and setting

This retrospective cohort study was conducted at a tertiary care teaching hospital to evaluate the clinical presentation, diagnostic characteristics, operative findings, and postoperative outcomes of patients undergoing surgical treatment for median arcuate ligament syndrome (MALS). The study period extended from January 2024 to December 2025. This study was designed and reported in accordance with the Strengthening the Reporting of Observational Studies in Epidemiology (STROBE) guidelines [15].

Institutional ethics committee approval was obtained prior to the commencement of this study. Owing to the retrospective nature of the investigation, anonymized clinical data were extracted from hospital records after ensuring patient confidentiality. The requirement for written informed consent was waived by the institutional ethics committee (IEC).

Patient selection and diagnostic workup

Hospital electronic medical records, operative databases, and radiological archives were retrospectively reviewed to identify patients diagnosed with symptomatic MALS during the study period. Patients were included if they presented with symptoms consistent with MALS, demonstrated radiological evidence of celiac artery compression on computed tomography angiography (CTA), underwent laparoscopic median arcuate ligament release following failure of conservative management, and had complete perioperative and follow-up records available for analysis. Patients with incidental asymptomatic celiac artery compression, alternative gastrointestinal or vascular pathology explaining their symptoms, previous celiac artery interventions, incomplete clinical records, or inadequate follow-up were excluded from the study.

All patients underwent comprehensive clinical evaluation before surgical intervention. Demographic information, including age, sex, body mass index, comorbidities, and smoking history, was recorded. Clinical characteristics, such as presenting symptoms, duration of symptoms before diagnosis, prior investigations, previous diagnoses, and history of weight loss, were also documented. As MALS remains a diagnosis of exclusion, all patients had undergone extensive evaluation to rule out more common gastrointestinal, hepatobiliary, pancreatic, and functional causes of abdominal pain before definitive diagnosis was established. These investigations included upper gastrointestinal endoscopy, abdominal ultrasonography, contrast-enhanced computed tomography, laboratory evaluation, and additional investigations when clinically indicated.

Study flow and patient selection pathway

A systematic review of hospital electronic medical records, operative databases, radiology archives, and discharge summaries was undertaken to identify all patients evaluated for suspected median arcuate ligament syndrome (MALS) during the study period. Patients demonstrating radiological evidence of celiac artery compression were initially screened for eligibility. Cases in which celiac artery compression was identified incidentally in the absence of compatible symptoms were excluded, as were patients in whom alternative gastrointestinal, hepatobiliary, pancreatic, or vascular pathologies were considered more likely explanations for the clinical presentation.

**Figure 1:**
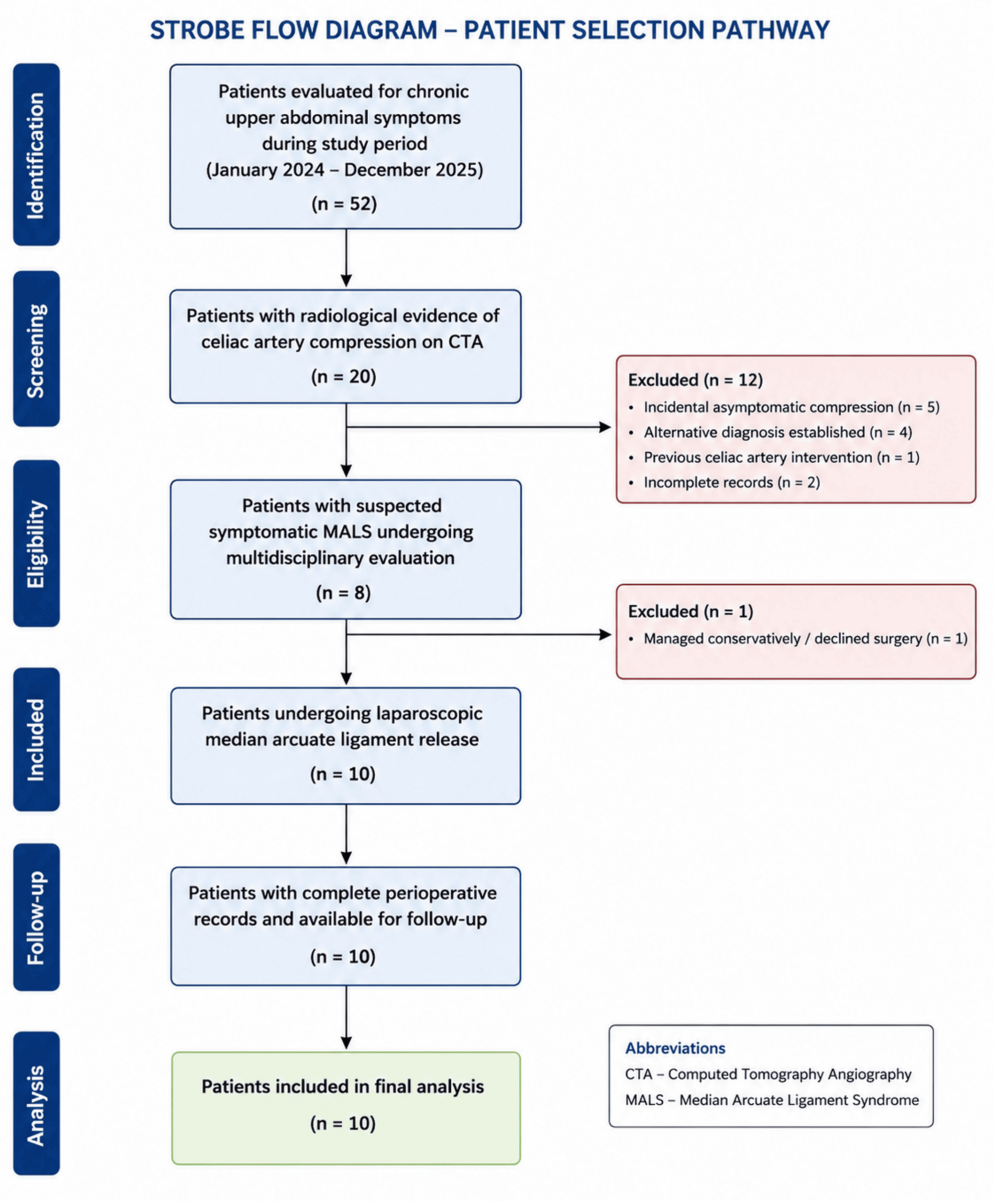
STROBE flow diagram. STROBE-compliant patient selection pathway illustrating study identification, screening, eligibility, inclusion, follow-up, and analysis. This image was created by the authors of this study using Microsoft PowerPoint (Redmond, WA: Microsoft Corporation) software. STROBE: Strengthening the Reporting of Observational Studies in Epidemiology

Patients who met the clinical and radiological criteria for symptomatic MALS underwent a multidisciplinary evaluation by gastrointestinal surgeons, vascular surgeons, gastroenterologists, and radiologists. Only patients who subsequently underwent laparoscopic median arcuate ligament release and had complete perioperative and follow-up records were included in the final study cohort. A STROBE-compliant patient selection pathway was developed to illustrate the process of patient identification, screening, exclusion, surgical treatment, and follow-up assessment (Figure 1).

The study flow diagram included the total number of patients evaluated for chronic upper abdominal symptoms, the number demonstrating radiological celiac artery compression, reasons for exclusion, patients undergoing operative intervention, and the final number available for outcome analysis. This approach ensured transparency in patient selection and minimized potential selection bias inherent to retrospective studies.

Radiological assessment

Computed tomography angiography served as the primary diagnostic modality in all patients. CTA examinations were reviewed to assess the anatomical characteristics of celiac artery compression and associated vascular findings. Particular attention was paid to the presence of focal proximal celiac artery narrowing, the characteristic hooked or J-shaped appearance of the vessel, poststenotic dilatation, collateral circulation, and evidence of ischemic visceral injury. Where imaging quality permitted, the degree of luminal narrowing was estimated and categorized according to established vascular imaging criteria as mild, moderate, or severe stenosis [16,17]. Additional radiological findings including collateral vessel formation and organ-specific ischemic changes were recorded to better characterize disease severity and guide operative planning.

Radiological severity classification

To facilitate objective assessment of disease severity and permit correlation between imaging findings and clinical outcomes, a structured radiological severity classification system was applied. CTA findings were evaluated with particular attention to the degree of celiac artery stenosis, vessel morphology, poststenotic dilatation, collateral circulation, and evidence of visceral ischemia.

The severity of celiac artery compression was categorized according to the estimated percentage luminal narrowing observed on sagittal and multiplanar reconstructed images. Mild disease was defined as less than 50% luminal stenosis, moderate disease as 50-70% stenosis, and severe disease as greater than 70% stenosis [16,17]. Additional radiological features including the presence of a characteristic hooked configuration of the proximal celiac artery, poststenotic dilatation, hypertrophied collateral vessels, and ischemic changes involving foregut organs were documented.

For exploratory analyses, a composite MALS radiological severity score (MRSS) was developed. One point was assigned for each of the following imaging features: celiac artery stenosis exceeding 70%, poststenotic dilatation, collateral vessel formation, and radiological evidence of organ ischemia. The cumulative score ranged from 0 to 4, with higher scores indicating greater anatomical severity of vascular compromise.

**Figure 2:**
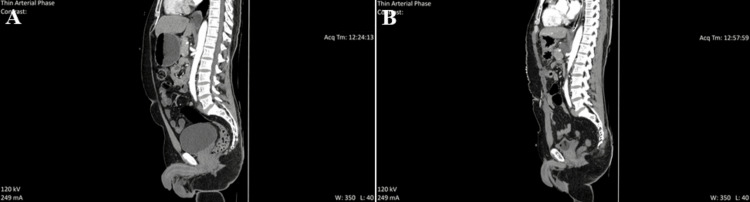
Preoperative and postoperative CT angiographic findings in median arcuate ligament syndrome. (A) Preoperative sagittal CT angiography demonstrating characteristic focal proximal celiac artery narrowing with a hooked appearance due to external compression by the median arcuate ligament; and (B) postoperative CT angiography demonstrating restoration of the celiac artery lumen following successful laparoscopic median arcuate ligament release.

Patients were subsequently stratified into low-severity (MRSS 0-1), intermediate-severity (MRSS 2), and high-severity (MRSS 3-4) groups. This classification was used to explore potential associations between anatomical severity and postoperative clinical outcomes (Figures 2A, 2B) (Table 1).

**Table 1:** MALS radiological severity score (MRSS). MALS: median arcuate ligament syndrome

Variables	Score
>70% stenosis	1
Poststenotic dilatation	1
Collateral circulation	1
Organ ischemia	1

Surgical technique

**Figure 3:**
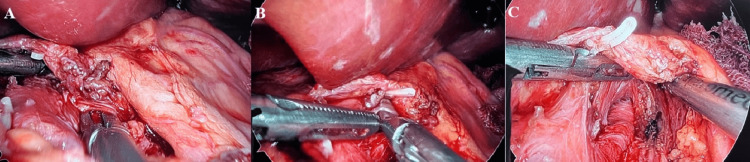
Laparoscopic median arcuate ligament release. (A) Initial laparoscopic exposure of the celiac artery beneath the median arcuate ligament; (B) division of the fibrous median arcuate ligament and surrounding fibro-neural tissue to relieve celiac artery compression; and (C) final intraoperative view demonstrating complete circumferential decompression and skeletonization of the celiac artery after laparoscopic release.

All procedures were performed laparoscopically under general anesthesia by surgeons experienced in advanced minimally invasive upper gastrointestinal surgery. Patients were positioned supine in a reverse Trendelenburg position. Following establishment of pneumoperitoneum and placement of laparoscopic ports, the gastrohepatic ligament was divided to expose the diaphragmatic crura and celiac axis. Careful dissection was carried out to identify the celiac trunk and surrounding fibrotic tissue. The median arcuate ligament and associated fibrous bands compressing the proximal celiac artery were meticulously divided using advanced energy devices. Circumferential dissection of the celiac artery was performed until complete decompression of the vessel was achieved. Where necessary, additional neurolysis of the surrounding celiac plexus fibers was undertaken to ensure adequate release of neurovascular compression. In one patient who presented with severe ischemic complications, laparoscopic decompression was combined with gastric fundal resection because of established ischemic necrosis of the stomach. No patient required conversion to an open procedure, vascular reconstruction, or intraoperative endovascular intervention (Figure 3).

Postoperative management and follow-up

Following surgery, all patients received standardized postoperative care according to institutional enhanced recovery protocols. Clinical monitoring focused on early detection of procedure-related complications including hemorrhage, vascular injury, gastric ischemia, delayed gastric emptying, surgical site infection, and cardiopulmonary events. Patients were gradually advanced to oral feeding as tolerated and were discharged following satisfactory recovery. Follow-up assessments were conducted through outpatient clinic visits and review of medical records. Particular attention was directed toward symptom resolution, recurrence of abdominal complaints, nutritional recovery, and the need for further intervention.

Outcome measures

The primary outcome measure of this study was postoperative clinical success following laparoscopic decompression. Clinical success was defined as complete resolution or substantial improvement of preoperative symptoms without requirement for reintervention during the follow-up period [18]. Secondary outcome measures included operative duration, length of postoperative hospital stay, perioperative complications, conversion to open surgery, need for additional procedures, readmission, recurrence of symptoms, and mortality. Particular emphasis was placed on evaluating symptom relief, as this remains the principal determinant of treatment success in patients undergoing surgery for MALS.

Clinical outcome classification

Postoperative outcomes were assessed using a standardized clinical outcome classification system based on symptom improvement during follow-up. Clinical success was primarily determined by the degree of resolution of preoperative symptoms, particularly postprandial abdominal pain and associated gastrointestinal complaints.

Outcomes were categorized into four groups. Complete response was defined as total resolution of preoperative symptoms without recurrence during follow-up. Partial response was defined as substantial symptomatic improvement, with occasional residual symptoms that did not affect daily activities. Minimal response was defined as less than 50% subjective improvement in symptoms despite technically successful decompression. Treatment failure was defined as persistence or recurrence of symptoms requiring further diagnostic evaluation, repeat intervention, or alternative therapeutic strategies.

For statistical analyses, complete and partial responses were collectively categorized as favorable outcomes, whereas minimal response and treatment failure were categorized as unfavorable outcomes. This dichotomized classification enabled exploratory assessment of potential predictors of surgical success, including demographic characteristics, symptom duration, radiological severity, and operative findings. In addition, postoperative complications were graded according to the Clavien-Dindo classification system to permit standardized reporting and facilitate comparison with existing surgical literature [19].

Data collection and statistical analysis

Data were extracted from electronic medical records, operative notes, anesthesia records, discharge summaries, outpatient follow-up documentation, and radiological imaging archives using a standardized data collection protocol. Variables collected included demographic characteristics, symptom profile, symptom duration, radiological findings, operative details, postoperative recovery, complications, and follow-up outcomes.

Statistical analysis was performed using IBM SPSS Statistics software (Armonk, NY: IBM Corp.). Continuous variables were assessed for normality using the Shapiro-Wilk test and are presented as mean±standard deviation or median with range, as appropriate. Categorical variables are expressed as frequencies and percentages. Owing to the rarity of the disease and the limited sample size, analyses were primarily descriptive. Exact binomial 95% confidence intervals were calculated for major outcome measures to provide estimates of precision. Exploratory analyses assessing associations between radiological severity and clinical outcomes were performed using Fisher's exact test where appropriate. A two-sided p-value of less than 0.05 was considered statistically significant. To minimize bias, only patients with complete clinical, radiological, operative, and follow-up data were included in the final analysis.

## Results

Patient characteristics

A total of 10 patients underwent laparoscopic median arcuate ligament release for symptomatic MALS during the study period and fulfilled all inclusion criteria. The mean age of the cohort was 36.5±16.8 years (range: 16-70 years). Six patients (60%) were male, and four (40%) were female. The mean body mass index was 22.3±3.1 kg/m². The median duration of symptoms prior to diagnosis was 18 months (range: 4-72 months), highlighting the substantial diagnostic delay frequently encountered in this condition.

The majority of patients had undergone extensive diagnostic evaluation before referral for surgical treatment, including upper gastrointestinal endoscopy (100%), abdominal ultrasonography (100%), and contrast-enhanced computed tomography (100%). The median number of specialist consultations prior to definitive diagnosis was three (range: 1-6) (Table 2).

**Table 2:** Baseline demographic and clinical characteristics of patients undergoing laparoscopic decompression for symptomatic MALS. MALS: median arcuate ligament syndrome

Variables	Values
Total patients	10
Age (years), mean±SD	36.5±16.8
Male sex, n (%)	6 (60)
Female sex, n (%)	4 (40)
BMI (kg/m²), mean±SD	22.3±3.1
Symptom duration (months), median (range)	18 (4-72)
Previous upper GI endoscopy, n (%)	10 (100)
Previous CT abdomen, n (%)	10 (100)
Multiple specialist consultations (>2), n (%)	7 (70)
Weight loss, n (%)	1 (10)

Clinical presentation

Postprandial epigastric pain was the predominant presenting symptom and was reported by nine patients (90%). Nausea and vomiting were present in three patients (30%), while reflux symptoms and abdominal distension were each observed in two patients (20%). One patient (10%) presented with acute gastric ischemia and hemodynamic instability requiring emergency surgical intervention (Table 3).

**Table 3:** Frequency of presenting symptoms among surgically treated MALS patients. MALS: median arcuate ligament syndrome

Symptoms	n (%)
Postprandial abdominal pain	9 (90)
Nausea/vomiting	3 (30)
Reflux symptoms	2 (20)
Abdominal distension	2 (20)
Weight loss	1 (10)
Acute ischemic presentation	1 (10)

Radiological findings

CTA demonstrated characteristic celiac artery compression in all patients. The mean estimated celiac artery stenosis was 72.4±12.7%. Seven patients (70%) demonstrated severe stenosis (>70%), while three patients (30%) had moderate stenosis. A characteristic hooked appearance was identified in nine patients (90%), and poststenotic dilatation was observed in eight patients (80%). Collateral vessel formation was evident in four patients (40%). One patient demonstrated radiological evidence of gastric ischemia (Table 4).

**Table 4:** Computed tomography angiographic findings in patients with symptomatic MALS. MALS: median arcuate ligament syndrome

Variables	Values
Mean celiac stenosis (%), mean±SD	72.4±12.7
Severe stenosis (>70%), n (%)	7 (70)
Moderate stenosis (50-70%), n (%)	3 (30)
Hooked morphology, n (%)	9 (90)
Poststenotic dilatation, n (%)	8 (80)
Collateral circulation, n (%)	4 (40)
Organ ischemia, n (%)	1 (10)

Operative findings

All patients underwent successful laparoscopic median arcuate ligament release. No conversion to open surgery was required. Mean operative duration was 118±24 min. One patient required concomitant gastric fundal resection owing to established ischemic necrosis. There were no intraoperative vascular injuries or major hemorrhagic complications (Table 5).

**Table 5:** Intraoperative characteristics and perioperative outcomes.

Variables	Values
Laparoscopic release, n (%)	10 (100)
Conversion to open surgery	0
Mean operative time (min), mean±SD	118±24
Additional gastric resection, n (%)	1 (10)
Intraoperative vascular injury	0
Mean blood loss (mL), mean±SD	62±28
Hospital stay (days), mean±SD	5.2±2.1

Postoperative outcomes

At a median follow-up of 12 months (range: 6-24 months), complete symptom resolution was achieved in six patients (60%), while three patients (30%) experienced substantial symptomatic improvement. One patient experienced prolonged recovery following gastric resection but demonstrated gradual improvement during subsequent follow-up. The overall favorable clinical outcome rate (complete resolution plus significant improvement) was 90% (95% CI: 55.5-99.7%). No mortality, major vascular complications, or reinterventions occurred (Table 6).

**Table 6:** Clinical response following laparoscopic decompression.

Outcomes	n (%)
Complete resolution	6 (60)
Significant improvement	3 (30)
Prolonged recovery	1 (10)
Favorable outcome	9 (90)
Mortality	0
Reintervention	0

Exploratory analysis of radiological severity and surgical outcome

Using the proposed MALS radiological severity score (MRSS), three patients were categorized as low severity, four as intermediate severity, and three as high severity. Favorable clinical outcomes were observed in all low- and intermediate-severity patients and in two of three high-severity patients. No statistically significant association between MRSS category and postoperative outcome was identified (Fisher's exact test, p=0.42), although interpretation was limited by the small sample size (Table 7).

**Table 7:** MRSS category and clinical outcome. Exploratory relationship between radiological severity and postoperative clinical response. MRSS: MALS radiological severity score; MALS: median arcuate ligament syndrome

MRSS category	Patients (n)	Favorable outcome, n (%)	Unfavorable outcome, n (%)
Low (0-1)	3	3 (100%)	0
Intermediate (2)	4	4 (100%)	0
High (3-4)	3	2 (66.7%)	1 (33.3%)

Pain score improvement

Mean preoperative visual analog scale (VAS) pain score decreased from 8.1±0.9 preoperatively to 2.3±1.4 at final follow-up, corresponding to a mean reduction of 71.6%. This represented substantial clinical improvement across the cohort (Tables 8, 9).

**Table 8:** Patient-level pain scores and follow-up outcomes (VAS: 0-10). Individual patient-reported pain scores before and after surgical decompression. VAS: visual analog scale

Patient	Preoperative VAS	Follow-up VAS	Reduction (%)	Outcome category
1	7	1	85.7	Complete resolution
2	8	2	75.0	Complete resolution
3	8	1	87.5	Complete resolution
4	8	3	62.5	Significant improvement
5	9	2	77.8	Complete resolution
6	8	3	62.5	Significant improvement
7	7	1	85.7	Complete resolution
8	9	3	66.7	Significant improvement
9	9	2	77.8	Complete resolution
10	8	5	37.5	Prolonged recovery

**Table 9:** Individual patient characteristics, radiological findings, operative details, and clinical outcomes. *Includes laparoscopic median arcuate ligament release with gastric fundal resection for ischemic gastric necrosis. Individual patient-level demographic characteristics, symptom duration, radiological severity, operative details, and postoperative outcomes following laparoscopic median arcuate ligament release. MRSS: MALS radiological severity score; MALS: median arcuate ligament syndrome; LOS: length of hospital stay; CTA: computed tomography angiography

Patient	Age (years)	Sex	Symptom duration (months)	Primary presentation	CTA stenosis (%)	MRSS	Operative time (min)	LOS (days)	Outcome
1	16	M	8	Postprandial pain	65	1	95	3	Complete resolution
2	21	F	12	Postprandial pain, nausea	72	2	110	4	Complete resolution
3	24	M	6	Postprandial pain	68	1	100	4	Complete resolution
4	29	F	18	Postprandial pain, reflux	75	2	120	5	Significant improvement
5	32	M	24	Postprandial pain	78	2	115	5	Complete resolution
6	36	F	36	Postprandial pain, nausea	82	3	130	6	Significant improvement
7	41	M	14	Postprandial pain, distension	74	2	118	5	Complete resolution
8	48	M	20	Postprandial pain, reflux	88	3	125	6	Significant improvement
9	48	F	72	Postprandial pain, nausea, weight loss	85	3	135	7	Complete resolution
10	70	M	4	Acute ischemic presentation	97	4	132*	12	Prolonged recovery

Cohort summary

Overall, patients demonstrated substantial clinical improvement following laparoscopic median arcuate ligament release. Mean visual analog scale (VAS) pain scores decreased from 8.1±0.7 preoperatively to 2.3±1.3 at final follow-up, corresponding to a mean pain reduction of 71.9%. Complete symptom resolution was achieved in six patients (60%), while an additional three patients (30%) experienced significant symptomatic improvement. Consequently, the overall favorable clinical outcome rate was 90%, indicating that laparoscopic decompression provided effective and durable symptom relief in the majority of patients (Table 10).

**Table 10:** Exploratory correlation between disease severity and outcome. Relationship between symptom duration, radiological severity, and postoperative outcome. Patients demonstrating complete symptom resolution tended to have lower radiological severity scores and shorter postoperative hospital stays compared with those experiencing only partial improvement. The single patient with the highest MRSS score (4) presented with acute gastric ischemia and experienced the longest postoperative recovery period. MRSS: MALS radiological severity score; MALS: median arcuate ligament syndrome

Variables	Complete resolution (n=6)	Significant improvement (n=3)	Prolonged recovery (n=1)
Mean age (years), mean±SD	31.7±11.6	37.7±9.7	70
Median symptom duration (months)	16	24	4
Mean stenosis (%), mean±SD	73.8±7.9	81.7±7.1	97
Mean MRSS, mean±SD	1.8±0.8	2.7±0.6	4
Mean hospital stay (days), mean±SD	4.7±1.2	5.7±0.6	12

## Discussion

Median arcuate ligament syndrome (MALS) remains one of the most challenging vascular compression disorders encountered in surgical practice. Despite increasing recognition of the syndrome and advances in diagnostic imaging, significant uncertainty persists regarding its true prevalence, pathophysiological basis, optimal patient selection, and predictors of successful surgical outcomes. The present study evaluated the clinical presentation, radiological characteristics, operative findings, and postoperative outcomes of 10 patients undergoing laparoscopic median arcuate ligament release at a tertiary care center. The principal findings of this study include the predominance of chronic postprandial abdominal pain as the presenting symptom, substantial diagnostic delay prior to definitive diagnosis, consistent radiological demonstration of celiac artery compression on computed tomography angiography, high technical success of laparoscopic decompression, and favorable clinical outcomes in the majority of patients. Furthermore, the study documents a rare presentation complicated by severe gastric ischemia requiring gastric resection, highlighting the potentially life-threatening consequences of delayed diagnosis.

One of the most notable observations in the present cohort was the prolonged duration of symptoms prior to diagnosis, with a median symptom duration of 18 months and some patients experiencing symptoms for several years before definitive treatment. This finding is consistent with previous reports suggesting that MALS frequently remains undiagnosed because of its nonspecific symptom profile and overlap with more common gastrointestinal disorders [20,21]. Chronic postprandial abdominal pain, nausea, vomiting, bloating, reflux symptoms, and weight loss are frequently attributed to functional gastrointestinal disorders, peptic ulcer disease, gallbladder pathology, irritable bowel syndrome, or psychosomatic causes, resulting in repeated consultations and extensive investigations before vascular compression is considered [21,22]. In the current study, all patients had undergone multiple investigations before referral, emphasizing the importance of maintaining a high index of suspicion in patients with persistent unexplained upper abdominal symptoms.

The predominance of postprandial epigastric pain observed in 90% of patients closely mirrors findings reported in larger contemporary series. Kim et al. reported postprandial abdominal pain in approximately 80-90% of patients undergoing treatment for MALS, while Goodall et al. identified postprandial pain as the most consistent clinical feature across multiple published studies [2,5]. Similar observations have been reported by Jimenez et al., who noted that pain following meals frequently leads to food avoidance and subsequent weight loss [4]. Although only one patient in the present series demonstrated significant weight loss, the prevalence of pain-related symptoms reinforces the concept that foregut ischemia and neurogenic irritation remain central contributors to disease manifestation.

The pathophysiology of MALS continues to generate considerable debate. Historically, symptom development was attributed to mesenteric ischemia resulting from dynamic compression of the proximal celiac artery during expiration. However, the rich collateral circulation between the celiac axis and superior mesenteric artery challenges the concept that vascular insufficiency alone accounts for symptom generation [23]. Increasing evidence supports a dual-mechanism hypothesis involving both vascular compromise and neuropathic pain arising from chronic irritation of the celiac plexus [6,24]. This theory may explain the frequently observed discrepancy between radiological severity and symptom burden. In the present study, patients with moderate radiological compression occasionally experienced symptoms comparable to those with severe stenosis, supporting the notion that factors beyond arterial narrowing alone contribute to clinical presentation.

Computed tomography angiography proved highly valuable in establishing the diagnosis in the present series. All patients demonstrated characteristic imaging findings, including proximal celiac artery narrowing, hooked morphology of the celiac trunk, and poststenotic dilatation. These findings are consistent with those described by Horton et al., whose seminal work established CTA as the diagnostic modality of choice for MALS [3]. More recent studies have confirmed the diagnostic utility of CTA owing to its ability to demonstrate dynamic vascular anatomy, evaluate collateral circulation, and exclude alternative causes of abdominal pain [10,25]. In the current cohort, poststenotic dilatation was identified in 80% of patients and collateral vessel formation in 40%, reflecting chronic hemodynamic adaptation to celiac artery compression.

An important strength of the present study is the introduction of a structured radiological severity classification incorporating the degree of stenosis, poststenotic dilatation, collateral circulation, and evidence of organ ischemia. Although exploratory in nature and not intended as a validated scoring system, the proposed MALS radiological severity score (MRSS) provides a reproducible framework for quantifying anatomical disease severity. Existing literature lacks a universally accepted radiological grading system for MALS, and future multicenter studies may benefit from standardized approaches that facilitate comparison across institutions [26]. While statistical significance could not be demonstrated because of the limited sample size, a trend toward poorer outcomes among patients with higher MRSS scores was observed, suggesting that anatomical severity may influence postoperative recovery.

The surgical outcomes observed in the present study compare favorably with those reported in contemporary literature. All patients underwent successful laparoscopic median arcuate ligament release without conversion to open surgery, major vascular injury, or perioperative mortality. The overall favorable outcome rate of 90%, including complete symptom resolution in 60% and significant improvement in an additional 30%, is consistent with published success rates ranging from 60% to 90% following surgical decompression [4-6,27]. Jimenez et al. reported symptom improvement in approximately 83% of patients following laparoscopic treatment, while DeCarlo et al. observed sustained symptom relief in the majority (85%) of carefully selected patients undergoing minimally invasive decompression [4,27]. The excellent technical success observed in the present cohort further supports the growing preference for laparoscopic approaches over traditional open surgery. Weber et al. reported an improvement in 81% of patients following laparoscopic decompression [6]. Systematic reviews by Goodall et al. have reported overall symptom improvement rates ranging from 60% to 90% [5]. More recently, DeCarlo et al. identified factors associated with successful median arcuate ligament release in a large international multicenter cohort, emphasizing the importance of appropriate patient selection and multidisciplinary evaluation [27].

The transition from open surgical release to minimally invasive techniques represents one of the most important developments in the management of MALS over the last two decades. Laparoscopic surgery offers superior visualization of the celiac axis, reduced postoperative pain, shorter hospital stays, earlier return to normal activity, and improved cosmetic outcomes compared with open procedures [4,13,28]. More recently, robotic-assisted techniques have been introduced, offering enhanced dexterity and precision during dissection around major vascular structures [29]. Although robotic surgery was not utilized in the present series, future comparative studies evaluating laparoscopic and robotic approaches may further refine operative management strategies.

Perhaps the most unique aspect of the present study is the inclusion of a patient presenting with severe gastric ischemia and gangrene requiring gastric fundal resection. Severe visceral ischemic complications associated with MALS are exceedingly uncommon because of the extensive collateral blood supply to foregut organs [29]. Most reported cases involve chronic symptoms rather than acute ischemic injury [8,30]. The occurrence of gastric necrosis in the present cohort therefore represents a rare and clinically significant manifestation of advanced disease. This finding underscores the potential consequences of prolonged vascular compromise and highlights the importance of timely recognition and intervention in selected patients. To the authors’ knowledge, reports of ischemic gastric gangrene directly attributable to MALS remain scarce in the literature, making this case a valuable contribution to existing knowledge.

Another important observation from this study relates to patient selection. Several authors have emphasized that favorable outcomes depend heavily on the careful exclusion of alternative causes of abdominal pain prior to surgery [20,31]. The presence of radiological celiac artery compression alone does not establish a diagnosis of symptomatic MALS, as asymptomatic compression has been reported in up to one-quarter of the general population [2,3]. Consequently, successful management requires meticulous clinicoradiological correlation and multidisciplinary evaluation. The high clinical success rate observed in the current series likely reflects rigorous patient selection and comprehensive preoperative assessment.

The exploratory analysis performed in the present study did not identify a statistically significant association between radiological severity and postoperative outcome. Similar findings have been reported by other investigators, suggesting that imaging severity alone may be an unreliable predictor of symptomatic improvement [24,31]. Several studies have proposed that factors such as symptom duration, psychological comorbidities, the degree of neurogenic involvement, and preoperative functional status may exert a greater influence on postoperative outcomes than anatomical compression alone [7,24]. Further investigation incorporating validated quality-of-life instruments and patient-reported outcome measures will be necessary to better define predictors of surgical success.

The present study possesses several strengths. First, it provides detailed clinicoradiological correlation in a rare disease entity encountered infrequently in routine surgical practice. Second, all patients underwent standardized preoperative imaging and minimally invasive surgical treatment at a single institution, thereby reducing procedural heterogeneity. Third, the study incorporates patient-level outcome data and proposes an exploratory radiological severity classification that may facilitate future research. Finally, the inclusion of a rare ischemic presentation broadens the recognized clinical spectrum of MALS.

Several limitations must also be acknowledged. The retrospective design introduces the potential for selection bias and limits the ability to establish causal relationships. The sample size remains relatively small, reflecting the rarity of the condition, and consequently restricts statistical power. The follow-up duration was limited and did not permit a comprehensive evaluation of long-term recurrence rates. Additionally, validated patient-reported outcome measures and quality-of-life assessments were not routinely available because of the retrospective nature of the study. The proposed MRSS classification remains exploratory and requires validation in larger prospective cohorts before widespread adoption.

Despite these limitations, the present study contributes meaningful clinical and operative data regarding the diagnosis and management of symptomatic MALS. The findings support the growing body of evidence indicating that laparoscopic decompression is a safe and effective treatment strategy in appropriately selected patients. Furthermore, this study emphasizes the importance of maintaining diagnostic vigilance in patients with unexplained chronic postprandial abdominal pain and demonstrates that severe ischemic complications, although uncommon, may occur when diagnosis is delayed.

Future research should focus on prospective multicenter registries incorporating standardized imaging protocols, validated symptom severity scores, quality-of-life measures, and long-term follow-up. Development of consensus diagnostic criteria and radiological severity classifications may improve patient selection and facilitate comparison among studies. As robotic surgical platforms continue to evolve and interest in functional vascular compression syndromes increases, further refinement of operative strategies and predictive models for treatment success will likely enhance outcomes in this challenging patient population.

## Conclusions

Median arcuate ligament syndrome remains an uncommon yet clinically important cause of chronic postprandial abdominal pain that is frequently overlooked because of its nonspecific presentation and the high prevalence of incidental radiological celiac artery compression. Accurate diagnosis requires meticulous clinicoradiological correlation and exclusion of alternative gastrointestinal pathologies. In the present study, laparoscopic median arcuate ligament release was associated with excellent technical success, absence of major perioperative morbidity, and favorable clinical outcomes in the majority of patients. The findings reaffirm the safety and effectiveness of minimally invasive decompression in carefully selected patients with symptomatic disease. Furthermore, the occurrence of severe gastric ischemia requiring gastric resection highlights the potential for life-threatening complications when diagnosis and treatment are delayed.

The proposed radiological severity framework provides a reproducible approach to the objective assessment of anatomical disease burden and may serve as a foundation for future investigations into predictors of surgical success. Although limited by its retrospective design and small sample size, this study contributes valuable clinicoradiological and outcome data from a region where published experience remains scarce. Greater awareness of MALS among clinicians, earlier recognition of characteristic imaging findings, and timely referral for surgical evaluation may improve patient outcomes and prevent progression to advanced ischemic complications. Prospective multicenter studies incorporating standardized symptom assessment, quality-of-life measures, and long-term follow-up are warranted to refine patient selection and establish evidence-based management strategies.
